# Cherry Valley Duck Galectin-2 Plays an Essential Role in Avian Pathogenic *Escherichia coli* Infection-Induced Innate Immune Response

**DOI:** 10.3389/fvets.2020.564088

**Published:** 2020-09-29

**Authors:** Tianxu Li, Hongyu Weng, Jing Lin, Tingting Zhang, Huihui Zhang, Xingdong Song, Xiaolan Hou, Liangmeng Wei

**Affiliations:** ^1^College of Animal Science and Veterinary Medicine, Sino-German Cooperative Research Centre for Zoonosis of Animal Origin of Shandong Province, Shandong Provincial Key Laboratory of Animal Biotechnology and Disease Control and Prevention, Shandong Provincial Engineering Technology Research Center of Animal Disease Control and Prevention, Shandong Agricultural University, Tai'an City, China; ^2^Collaborative Innovation Center for the Origin and Control of Emerging Infectious Diseases, Shandong First Medical University, Tai'an City, China

**Keywords:** Cherry Valley duck, Galectin-2, avian pathogenic *Escherichia coli*, antibacterial ability, innate immunity

## Abstract

Galectins play important roles in the host's innate immunity as pattern recognition receptors. In this study, the coding sequences of galectin-2 were identified from Cherry Valley ducks. Tissue distribution of duck galectin-2 (duGal-2) in healthy ducks and ducks infected with avian pathogenic Escherichia coli (APEC) was studied, respectively. The results showed that duGal-2 expression was higher in the gut, kidney, and liver tissue, and weakly expressed in the lung and brain, in healthy ducks; however, the expression level of duGal-2 was detected as being up-regulated after infection with APEC. In addition, knockdown or overexpression of duGal-2 in DEFs was achieved by small interference RNA (siRNA) transfection and plasmid transduction, respectively. The knockdown of duGal-2 led to a decrease in the expression of some inflammatory cytokines such as IL-1β, IL-6, and IL-8, while the expression levels of anti-inflammatory factor IL-10 were up-regulated. At the same time, the bacterial load of APEC was increased after knockdown of duGal-2 *in vitro*. However, the opposite results were obtained in the duGal-2 overexpression group. Taken together, duGal-2 plays an important role in the host against APEC infection.

## Introduction

Pattern recognition receptors (PRR) are responsible for identifying the pathogen-associated molecular patterns (PAMPs), and activating the innate immune response mainly includes the production of type I interferons (IFNs) and inflammatory cytokines (1), which is important for resisting infection by pathogenic microorganisms. Lectin is a protein that is widely distributed in animals, plants, and microorganisms. It selectively recognizes and non-covalently binds to sugar structures (2). Galectins (S-type lectins) are evolutionarily conservative and belong to the lectin superfamily, characterized by an affinity for β-galactosides and conserved carbohydrate recognition domains (CRDs) (3, 4). In mammals, it was found that galectins were widely distributed in immune-related organs and cells, suggesting that they are related to growth and immune function (5). Galectins are multifunctional molecules involved in cell adhesion, apoptosis (6), mRNA splicing (7), inflammatory reaction (8), and antitumor immune response (9). Most importantly, galectins play an essential role in innate immunity by recognizing PAMPs (10). So far, 15 members of the galectin family have been identified in mammals (4). Most research focuses mainly on galectin-1 and galectin-3, while galectin-2 is less studied, especially in the field of waterfowl. In consideration of the fact that the expression and function of innate immune receptors varies among different species, such as the lack of retinoic acid-inducible gene I receptor in chickens, the duck melanoma differentiation-associated protein 5 receptors become an important receptor against influenza virus; this may be the cause of the differential susceptibility in chickens and ducks to influenza viruses (11, 12). We have focused on the innate immunity of waterfowl. In the early stage, we cloned Laboratory of Genetics and Physiology 2 receptors from ducks and geese and studied their role in innate immunity (13, 14). We recently cloned the duck galectin-1 gene and proved that it plays an important role in anti-duck plague virus infection (15). It is necessary to certify and characterize galectin-2 in ducks. This will help us develop a more comprehensive understanding of the innate immune system of waterfowl.

Avian colibacillosis is caused by Avian pathogenic *Escherichia coli* (APEC) (16). In particular, the continuous emergence of APEC's multi-drug resistance (MDR) and extensive drug-resistant strains has caused great concern worldwide (17). Ducks infected with APEC developed pericarditis, perihepatitis, and airsacculitis at all stages, and ducklings are the most susceptible (18, 19). In this study, we cloned the coding sequences (CDs) of the galectin-2 gene of Cherry Valley ducks for the first time. The tissue distribution of duck galectin-2 (duGal-2) in healthy ducks and ducks infected with APEC was detected, respectively. Furthermore, we used RNA interference and gene overexpression to determine the role of duGal-2 in anti-bacterial effects and inducing inflammatory cytokines. This will provide a theoretical basis and experimental basis for further understanding ducks' anti-bacterial innate immune response and the pathogenesis of inflammatory diseases.

## Materials and Methods

### Animals, Cells, and Bacteria

One-day-old healthy Cherry Valley ducks were purchased from a farm (Tai'an, China). Duck embryo fibroblasts (DEFs) were prepared from 11-day-old duck embryos and maintained in Dulbecco's Modified Eagle Medium (DMEM) (Gibco, Grand Island, NY, USA) supplemented with 10% fetal bovine serum (FBS) (Gibco, Grand Island, NY, USA). All incubations were performed in 5% CO_2_ at 37°C. The bacterial strain of APEC *O1:K1* used in this study was previously preserved by our laboratory. The bacteria were cultured in Luria-Burtani (LB) medium at 37°C and were shaken at 220 rpm for 18 h.

### Cloning and Bioinformatic Analysis of DuGal-2

Total RNA was extracted from duck spleen using an RNA isolater (Vazyme, Nan'jing, China). The obtained RNA was reverse transcribed to cDNA using a HiScriptRII One-Step RT-PCR kit (Vazyme, Nan'jing, China). DuGal-2 complete CDs were cloned by polymerase chain reaction (PCR), primers for the gene were designed based on the sequence in National Center for Biotechnology Information (NCBI) GenBank (Table 1). All primers used in this study were synthesized by the Invitrogen company (Guang'zhou, China). The PCR condition was as follows: 3 min at 95°C for initial denaturation; 35 cycles of 95°C for 15 s, 60°C for 15 s, 72°C for 30 s, and 10 min at 72°C for the final extension. The PCR products were sent to the Qingke Company (Qing'dao, China) for DNA sequencing. Sequences were analyzed using BLAST alignment (https://blast.ncbi.nlm.nih.gov/Blast.cgi) tools in the NCBI databases. The amino acid sequence of duGal-2 was analyzed by the SMART (http://smart.embl.de/) program. Multiple sequence alignment was performed using the ClustalX2 program and edited with the online tool Boxshade (https://embnet.vital-it.ch/software/BOX_form.html). Neighbor-joining phylogenetic trees were established using the MEGA-X software (20).

**Table 1:** Primer information table of this research.

**Primer name**	**Sequence (5^**′**^-3^**′**^)**	**Purpose**
jGalectin-2-F	TCGCTTCCTACCTGGTGACT	gene cloning
jGalectin-2-R	AGAAAGCAGAGCAGCTGGAG	gene cloning
qGalectin-2-F	CAGGGACAGCACTGTCAAGA	qRT-PCR
qGalectin-2-R	ATCCAGCTTGAAGGAGGTGA	qRT-PCR
qIL-1β-F	TCATCTTCTACCGCCTGGAC	qRT-PCR
qIL-1β-R	GTAGGTGGCGATGTTGACCT	qRT-PCR
qIL-6-F	TTCGACGAGGAGAAATGCTT	qRT-PCR
qIL-6-R	CCTTATCGTCGTTGCCAGAT	qRT-PCR
qIL-8-F	AAGTTCATCCACCCTAAATC	qRT-PCR
qIL-8-R	GCATCAGAATTGAGCTGAGC	qRT-PCR
qIL-10-F	GCCTCCACTTGTCTGACCTC	qRT-PCR
qIL-10-R	CCTCCATGTAGAACCGCATC	qRT-PCR
qTNF-α-F	GAAGGGAATGAACCCTCCTC	qRT-PCR
qTNF-α-R	CAGGTTGCTGCACATACACC	qRT-PCR
qβ-actin-F	GGTATCGGCAGCAGTCTTA	qRT-PCR
qβ-actin-R	TTCACAGAGGCGAGTAACTT	qRT-PCR

### Animal Experiments

Three healthy two-week-old ducks were euthanized and their heart, liver, spleen, lung, kidney, brain, epencephalon, brainstem, thymus, pancreas, bursa of Fabricius, trachea, esophagus, muscular stomach, glandular stomach, skin, muscle, duodenum, jejunum, ileum, cecum, and rectum were harvested for detection of duGal-2 tissue expression by qRT-PCR.

Forty healthy four-week-old ducks were randomly divided into two groups. The experimental group was inoculated into the right thoracic air sac with 0.1 mL APEC (3.0 × 10^8^ CFU/mL), while the control group was inoculated with normal saline at the same time (21). In each group, three ducks were euthanized per day from 1 to 3 days post-infection (dpi), and their liver, lung, and brain were collected for RNA isolation to detect duGal-2 and inflammatory cytokines via qRT-PCR. Subsequently, part of the tissue was serially diluted nine times at a 1:10 dilution with physiological saline solution after grinding for purposes of calculating the total bacterial count by the plate count method.

### Construction of Expression Plasmids and Transfection

Expression plasmid pEGFP-duGal-2 was obtained by cloning the CDs of duGal-2 into the vector pEGFP (Invitrogen). The plasmid construction was verified by sequencing. The DEFs were incubated overnight to achieve 80% confluent before transfection. Expression plasmid pEGFP-duGal-2 or empty vector were transfected into DEFs with the Lipofectamine 2000 reagent (Invitrogen, Carlsbad, CA, USA) following the instructions.

### SiRNA Interference

The duGal-2 siRNA and negative control RNA were designed and synthesized by the GenePharma Company (Shanghai, China). The sequences are shown in Table 2. duGal-2 siRNA or negative control siRNA was transfected into DEFs with the Lipofectamine RNAiMAX transfection reagent (Invitrogen, Carlsbad, CA, USA) following the instructions. After 36 h post-transfected (hpt), the efficiency of the siRNAs was measured by RT-qPCR.

**Table 2:** The sequences of pSi-RNA.

**pSiRNA**	**Sequence (5^**′**^-3^**′**^)**	**Positions**
pSi-NC (sense)	UUCUCCGAACGUGUCACGUTT	
pSi-NC (antisense)	ACGUGACACGUUAGAATT	
pSi-Gal-2-1 (sense)	GCUGAUGGCUUUGUCAUCATT	82
pSi-Gal-2-1 (antisense)	UGAUGACAAAGCCAUCAGCTT	
pSi-Gal-2-2 (sense)	UCACGGGAUGGCAACAGUUTT	175
pSi-Gal-2-2 (antisense)	AACUGUUGCCAUCCCGUGATT	
pSi-Gal-2-3 (sense)	GAGACAGUCACAUGUGCUUTT	209
pSi-Gal-2-3 (antisense)	AAGCACAUGUGACUGUCUCT	

**Table 3:** Reference sequences information of galectin-2.

**Species**	**GeneBank accession numbers**
*Macaca nemestrina*	XM_011712434.1
*Papio Anubis*	XM_003905510.4
*Macaca muLatta*	XM_001087063.4
*Macaca fascicuLaris*	XM_005567358.2
*Gorilla*	XM_004063437.2
*Homo*	NM_006498.3
*Rattus norvegicus*	NM_133599.1
*Mus muscuus*	NM_025622.3
*Equus caballus*	XM_014736921.2
*Orcinus orca*	XM_004279453.2
*Tursiops truncates*	XM_019943172.1
*Sus scrofa*	NM_001142842.1
*Ovis aries*	XM_027968085.1
*Pantholops hodgsonii*	XM_005955610.1
*Meleagris gallopavo*	XM_019614931.1
*Columba livia*	XM_005514675.3
*Falco cherrug*	XM_027815986.1
*Ficedula albicollis*	XM_005039775.1
*Taeniopygia guttata*	XM_012569142.1
*Canis lupus familiaris*	NM_001284467.1
*Anas platyrhynchos*	XM_013096223.3
*Cavia porcellus*	XM_013153161.2
*Geospiza fortis*	XM_005421829.1
*Gallus gallus*	XM_025155386.1
*Bos indicus*	XM_019960160.1
*Oreochromis niloticus*	XM_005468402.4
*Maylandia zebra*	XM_004576047.4

### Antibacterial Activity of DuGal-2

To study the antibacterial activity of duGal-2, after the overexpression or knockdown of duGal-2 DEFs were infected with 1 × 10^6^ CFU/mL APEC *O1:K1* for 3 h and then washed with PBS three times. The cells were cultured in DMEM with 10% FBS containing gentamicin (100 mg/mL) at 37°C for 3 h to kill the extracellular APEC. The cell lysates were collected for analysis of the total bacterial count by the plate count method.

### Quantitative Real-Time PCR (qRT-PCR)

Total RNA was prepared from tissues and DEFs and reverse-transcribed to cDNA using the above method. The duGal-2 primers (qgalectin-2 F/R) for qRT-PCR were designed by primer 3 Input (v. 0.4.0, http://bioinfo.ut.ee/primer3-0.4.0/) online software. The other primers used in this study were designed based on previously published primer sequences (11). All the sequences are shown in Table 1. QRT-PCR was carried out by the 7500 Fast Real-Time PCR System (Applied Bio-Systems, Foster City, CA, USA), using ChamQ SYBR qPCR Master Mix (Vazyme, Nan'jing, China). The reaction system was performed in 20 μL volumes. The condition was as follows: 30 s at 94°C for pre-denatured, 40 cycles of 94°C for 5 s, 60°C for 30 s. A dissociation stage was performed to verify the specificity of the PCR products. The relative expression of each target gene was analyzed by the 2^−ΔΔ*Ct*^ method using duck β-actin as the internal reference (22). At least three independent experiments were performed for each sample.

### Statistical Analysis

All data were represented as means ± standard deviations (SD). Student's *t*-test was used to determine the statistical significance of differences by the Graph Pad Prism 8.0.1 software (Graph Pad Software Inc., San Diego, CA, USA). *P* < 0.05 was considered statistically significant and *P* < 0.01 was highly significant.

## Results

### Molecular Cloning and Sequence Analysis of DuGal-2

**Figure 1:**
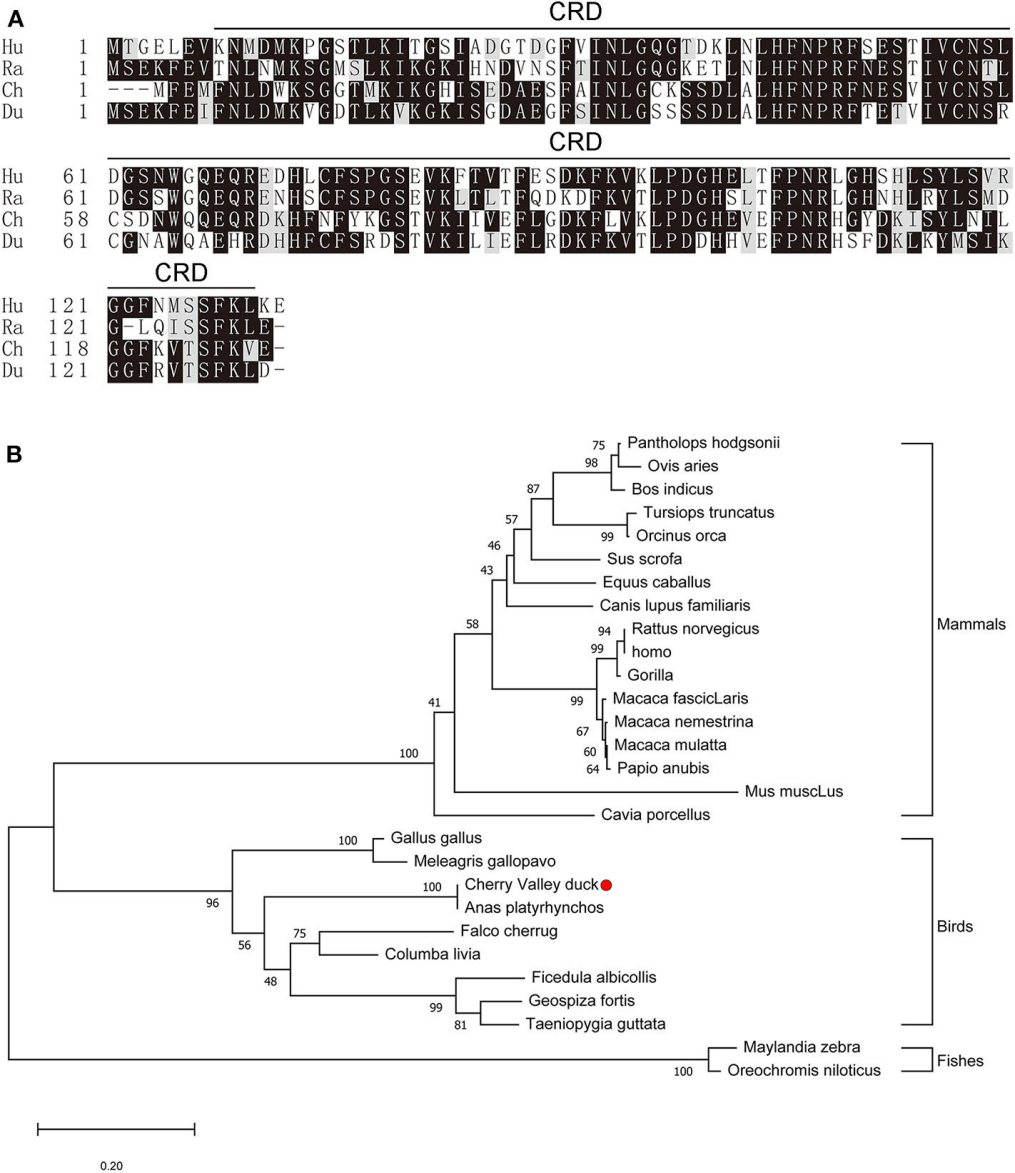
**(A)** Alignment of the deduced amino acid sequence of duGal-2 with other animals (Hu: Human, Ra: Rat, Ch: Chicken, Du: Cherry Valley Duck). Black shading indicates amino acid identity; gray shading indicates similarity (50% threshold). The predicted motifs for CRD are indicated with a black line on the alignment (AA 8-130). **(B)** The phylogenetic tree of the nucleic acid sequence of duGal-2 and other animals, a neighbor-joining tree was generated using MEGA-X and a 1000 bootstrap analysis was performed. The scale bar is 0.20. GenBank accession numbers are shown in Table 3.

The full-length CDs of duGal-2 contain 396 bp (GeneBank accession number: MT491431) and encodes 131 amino acid residues. Protein domains predicted using the SMART program show that duGal-2 has one CRD (Figure 1A) (AA 8-130). The phylogenetic result shows that duGal-2 has the closest relationship to mallard ducks (*anas platyrhynchos*) and was in the same subgroup as other birds; however, the galectin-2 of mammals or fishes belongs to two other subgroups, respectively (Figure 1B).

### Tissue Expression of DuGal-2

**Figure 2:**
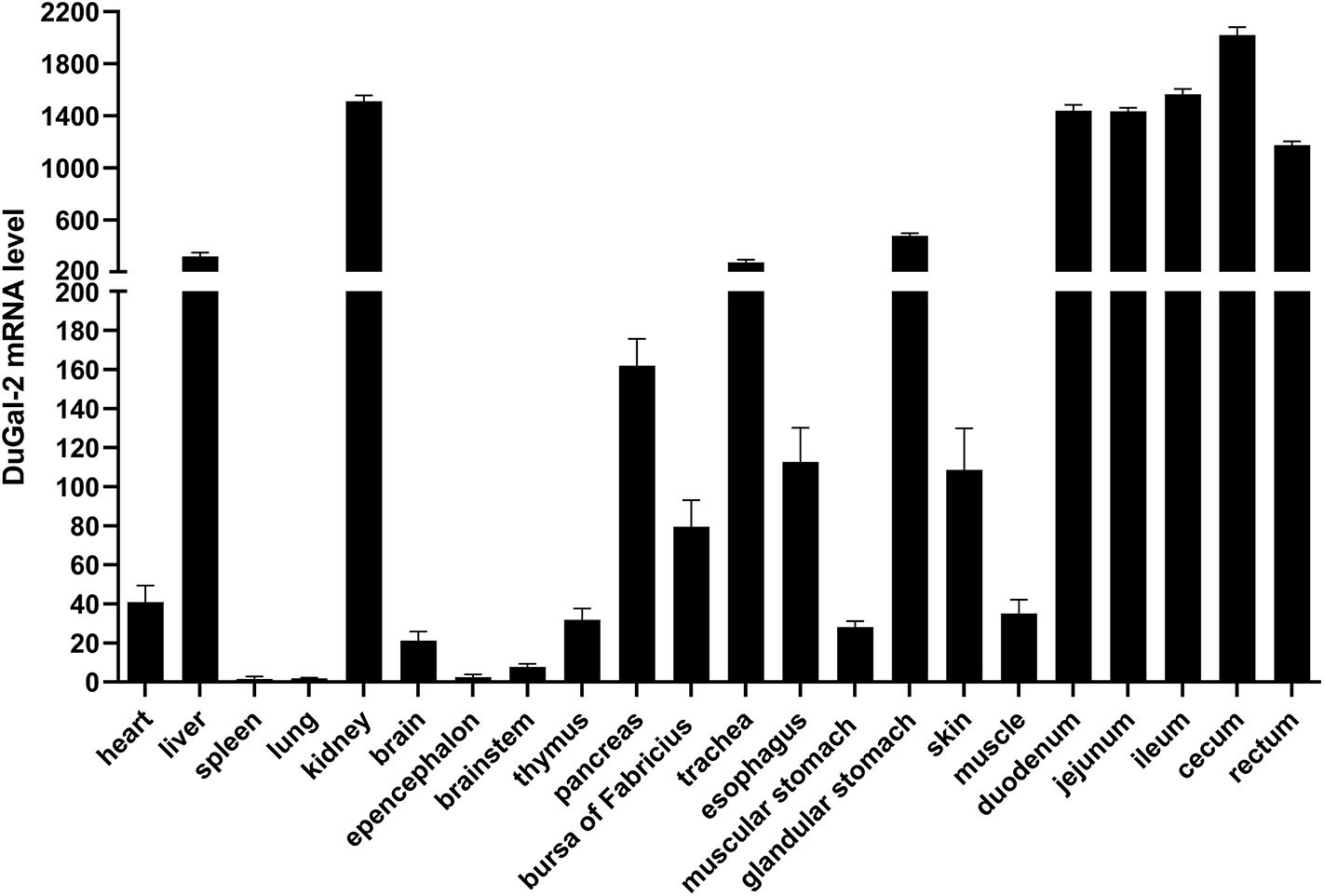
Tissue distribution of duGal-2 transcripts in healthy Cherry Valley duck. The relative mRNA levels were normalized to the expression of the β-actin gene from various tissues. Data were normalized to the spleen and error bars indicated SD.

The expression of duGal-2 mRNA in normal tissues was analyzed by qRT-PCR. The analysis results showed that the duGal-2 gene was expressed in all tissues tested. The spleen was chosen as standard tissue. The qRT-PCR results showed that the mRNA level of duGal-2 was higher in intestinal tract, kidney, and liver tissues, and lower in the lung and epencephalon (Figure 2). This suggests that duGal-2 may be extensively involved in immune responses.

### Expression of DuGal-2 in Ducks Infected With APEC

**Figure 3:**
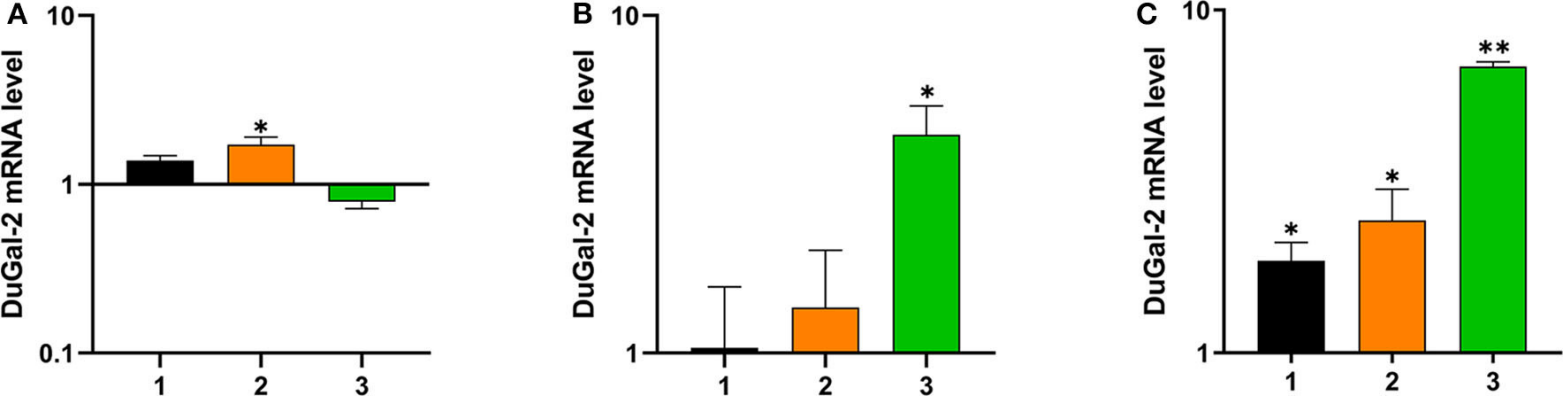
Relative expressions of duGal-2 at the mRNA level in liver **(A)**, lung **(B)**, and brain **(C)** after APEC infection. The fold change was calculated by the experimental duck vs. control duck at the same time point using the 2^−ΔΔCt^ method. All data are expressed as means ± SD (*n* = 3), and Student's *t*-tests were performed to evaluate the differences. *Significant difference (*P* < 0.05); **highly significant difference (*P* < 0.01).

To determine whether duGal-2 was involved in the host immunity against bacterial infection, the liver, lung, and brain tissue of ducks infected with APEC was collected and checked for the expression of duGal-2. QRT-PCR results showed that the expression level of duGal-2 was up-regulated at 1 and 2 (1.72-fold, *P* < 0.05) dpi, while it was down-regulated at 3 dpi in the liver (Figure 3A). In the lung, the expression level of duGal-2 gradually increased 1.03-, 1.37-, and 4.43-fold (*P* < 0.05) at 1, 2, and 3 dpi, respectively (Figure 3B). In the brain, the expression level of duGal-2 was significantly increased at 1, 2, and 3 dpi, with the highest level at 3 dpi, with 6.84-fold (*P* < 0.01) (Figure 3C). These results indicate that duGal-2 may be involved in the anti-bacterial immune response.

### DuGal-2 Knockdown Reduces Antibacterial Activity and Inflammatory Cytokine Production *in vitro*

**Figure 4:**
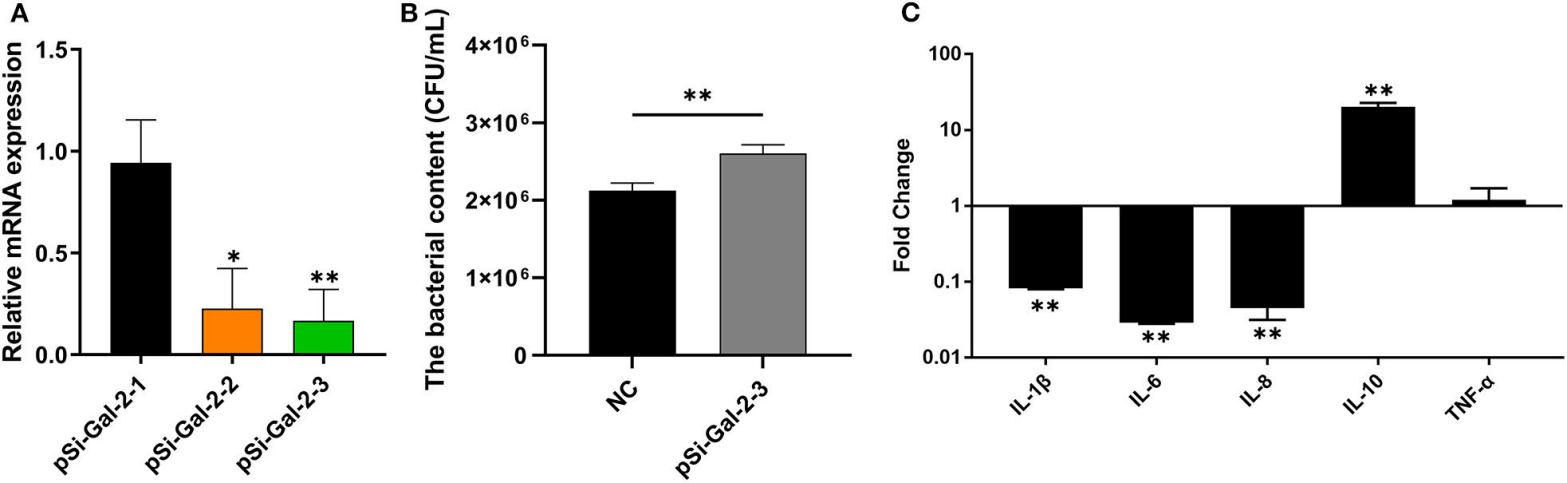
DuGal-2 knockdown decreases antibacterial activity and inflammatory cytokine production in DEFs cells. **(A)** Interference efficiency of pSi-Gal-2. **(B)** Intracellular bacterial CFU detection. **(C)** The detection of inflammatory cytokines. Data in **(A,B)** were analyzed using the 2^−ΔΔCt^ method. All data were expressed as means ± SD (*n* = 3), the ANOVA test **(A)** and the Student's *t*-test **(B,C)** were performed to evaluate differences. *Significant difference (*P* < 0.05); **Highly significant difference (*P* < 0.01).

To investigate the role of duGal-2 in anti-APEC immune response, the gene expression in DEFs was knocked down by RNA interference. Two SiRNA sequences (pSi-Gal-2-2/3) were able to knock down the mRNA level of duGal-2. However, the knockdown efficiency of pSi-Gal-2-3 was about 83% (*P* < 0.01), which was the most efficient. Thus, pSi-Gal-2-3 was used for further experiments (Figure 4A). After 36 hpt, cells were infected with APEC. The bacteria count and inflammatory cytokine were detected by the above method. The content of the duGal-2 knockdown group was apparently higher than that of the control group by about 20% (*P* < 0.01) (Figure 4B). The expression of IL-1β, IL-6, and IL-8 was significantly reduced as compared to the control group (*P* < 0.05). However, the anti-inflammatory factor IL-10 expression was observably up-regulated (*P* < 0.01),while the expression of TNF-α was not significantly different between the two groups after infection with APEC (Figure 4C). These results indicate that duGal-2 can induce the production of inflammatory factors and inhibit the growth of bacteria *in vitro*. Therefore, it is concluded that duGal-2 plays an important role in the innate immune response against APEC infection.

### Overexpression of DuGal-2 Promotes Antibacterial Activity and Inflammatory Cytokine Production *in vitro*

**Figure 5:**
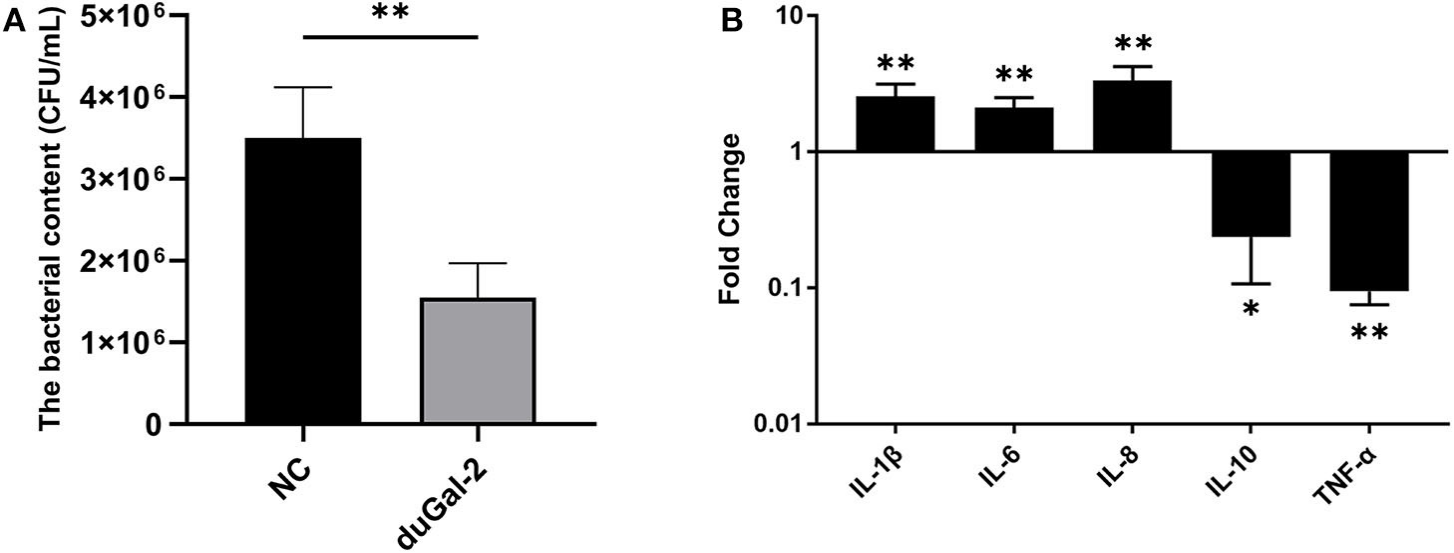
DuGal-2 overexpression promotes antibacterial activity and inflammatory cytokine production in DEFs cells. **(A)** Intracellular bacterial CFU detection. **(B)** The detection of inflammatory cytokines. The data in **(A,B)** were analyzed using the 2^−ΔΔCt^ method. All data are expressed as means ± SD (*n* = 3). Student's *t*-tests **(A,B)** were performed to evaluate differences. ^*^Significant difference (*P* < 0.05); ^**^Highly significant difference (*P* < 0.01).

DuGal-2 overexpression cells were obtained by transfection of pEGFP-duGal-2 plasmids. After 24 hpt, cells were infected with APEC. The bacteria count and inflammatory cytokine were detected by the above method. The content of the duGal-2 high expression group was significantly lower than that of the empty vector control group by about 66% (*P* < 0.01) (Figure 5A). The expression of IL-1β, IL-6, and IL-8 was statistically significantly up-regulated (*P* < 0.01), while the expression of IL-10 (*P* < 0.05) and TNF-α (*P* < 0.01) was observably reduced (Figure 5B).

## Discussion

At present, galectin-2 has been partially reported in mammals and fishes. In human medicine, galectin-2 was identified as a novel inhibitor of arteriogenesis. The regulation of galectin-2 may establish a new therapeutic strategy for the stimulation of arteriogenesis in patients with coronary artery disease (23). In mammals, galectin-2 and galectin-4 were reported to bind to intestinal epithelial cells and promote their restitution. Therefore, it is speculated that galectin-2 and galectin-4 play a beneficial role in the recovery of diseases characterized by epithelial barrier disruption (24). In addition, they demonstrated that galectin-2 induces apoptosis and ameliorates acute and chronic colitis in mice. Its wide dose-response range and lack of toxicity may serve as a new therapeutic agent in the treatment of inflammatory bowel disease (25). In aquatic animals, the recombinant galectin-2 protein from rock bream (*Oplegnathus fasciatus*) has the potential for hemagglutination and possessed an affinity for lactose and galactose. Furthermore, the recombinant protein can agglutinate and bind to potential pathogens and ciliates (26). Previous studies have shown that the galectin-2 of Nile tilapia (*Oreochromis niloticus*) performed agglutinating activities in response to both Gram-positive and Gram-negative bacteria (27). For now, the predicted sequence of galectin-2 in mallard ducks has been retrieved from NCBI. However, the sequence has not been cloned from ducks, and its expression in healthy duck and bacterial-infected duck tissues, as well as its participation in the host's innate immune response, have not been reported.

In this study, we analyzed the role of duGal-2 in the innate immune response induced by APEC infection and carried out the first systematic study of duGal-2. The full length of galectin-2 CDs was cloned from the spleens of Cherry Valley ducks for the first time. DuGal-2 CDs are composed of 396 bp encoding 131 amino acids. The duGal-2 sequence predicted by NCBI and sequence data obtained in this study had 100% matching sequences. The phylogenetic analysis showed that duGal-2 is more closely related to *anas platyrhynchos*, chickens (*gallus gallus*), and other birds than mammals or fishes.

To confirm the biological function of duGal-2, the tissue distribution of duGal-2 mRNA was detected in healthy ducks and ducks infected with APEC by qRT-PCR, respectively. As shown in Figure 2, duGal-2 was expressed in all tissues tested. It should be noted that the high expression of the gene in the intestinal tracts of healthy ducks. Previous studies have shown that the galectin-2 gene is related to the recognition of bacteria in fishes (26, 27), and the structure composed of galectin-2 and other galectins may help protect the gastrointestinal epithelial cells or tissues from an extreme luminal environment (including acidic gastric pH, bile, and pancreatic enzymes) (28, 29). The high expression of duGal-2 in the gut reminds us that it may have a similar function. Furthermore, the expression of duGal-2 was significantly up-regulated in the liver, lungs, and brains of ducks after infection with APEC. This suggests that galectin-2 may play an important role in the immune system during APEC infection.

To further determine whether duGal-2 has anti-bacterial ability, knockdown or overexpression of duGal-2 in DEFs was achieved by siRNA transfection and plasmid transduction, respectively. After infection with APEC, bacterial content was significantly up-regulated in the duGal-2 knockdown group (Figure 4B). In addition, expression levels of pro-inflammatory cytokine IL-1β, IL-6, and IL-8 were significantly inhibited, and the expression of anti-inflammatory factor IL-10 was more up-regulated than the pSi-NC group (Figure 4C). However, the opposite results were obtained in the duGal-2 overexpression group (Figure 5). According to the results of cytokine detection, it can be speculated that the inhibition of inflammatory cytokine expression may promote the proliferation capacity of APEC in DEFs. Previous studies have shown that the release of these cytokines, such as TNF-α, IL-6, and IL-1β, was induced by LPS during *Escherichia coli* infection (30, 31). Further, when Cherry Valley ducks are infected with certain viruses, such as the novel duck reovirus, duck tembusu virus, and duck plague virus, these factors will be increased (32, 33). The regulatory cytokines are also seen as being up-regulated during fungal infections (34). Our data show that duGal-2 can promote the production of these pro-inflammatory cytokines when the host is infected with APEC, and also suppresses the expression of IL-10, the anti-inflammatory factor, which further promotes the inflammatory response (35). This is important for survival from infection. However, overproduction of inflammatory cytokines may cause more serious consequences, even leading to cytokine storm, exacerbating the pathological process, and causing the death of the host (36).

In summary, we cloned and characterized duGal-2. Additionally, we phylogenetically analyzed duGal-2 with other species, detected its tissue expression, and studied its anti-bacterial ability.

## Data Availability Statement

The datasets presented in this study can be found in online repositories. The names of the repository/repositories and accession number(s) can be found here: https://www.ncbi.nlm.nih.gov/nuccore/MT491431.

## Ethics Statement

The animal study was reviewed and approved by Shandong Agricultural University Animal Care and Use Committee (No. SDAUA-2015-005).

## Author Contributions

TL and HW carried out the main experiments and wrote the manuscript. JL and TZ designed the experiments and analyzed the data. HZ, XS, and XH performed the experiment and wrote the discussion. LW reviewed the manuscript and approved the submission. All authors contributed to the article and approved the submitted version.

## Conflict of Interest

The authors declare that the research was conducted in the absence of any commercial or financial relationships that could be construed as a potential conflict of interest.
